# Household Contact Screening Adherence among Tuberculosis Patients in Northern Ethiopia

**DOI:** 10.1371/journal.pone.0125767

**Published:** 2015-05-08

**Authors:** Gebremedhin Berhe Gebregergs, Wondmu Gebeyehu Alemu

**Affiliations:** 1 Bahirdar University, College of Medicine and Health Sciences, School of Public Health, Bahir Dar, Ethiopia; 2 Amhara National Regional State Health Bureaus, Health Research and Technology Transfer Core Process, Bahir Dar, Ethiopia; Fundació Institut d’Investigació en Ciències de la Salut Germans Trias i Pujol. Universitat Autònoma de Barcelona SPAIN

## Abstract

**Background:**

Household contacts of active tuberculosis cases are at high risk of getting tuberculosis disease. Tuberculosis detection rate among contacts of household members is high. Hence, this study investigated household contact screening adherence and associated factors among tuberculosis patients in Amhara region, Ethiopia.

**Methods:**

A cross-sectional study was conducted from April 10 - June 30, 2013 in five urban districts of Amhara region, where 418 patients receiving treatment at tuberculosis clinic were interviewed. All patients were interviewed using structured and pre-tested questionnaire. Bringing at least one household contact to TB clinic was regarded as adherent to household contacts screening. Bivariate and multiple logistic regressions were used to investigate association.

**Results:**

The overall adherence to household contact screening in Amhara region was 33.7%. Adherence was higher among Muslims than Christians. Adherence was high if patient took health education from Health Care Worker [AOR: 3.22, 95% CI: 1.88 to 5.51] and 2.17 times higher if patient had sufficient knowledge on tuberculosis [AOR: 2.17, 95% CI: 1.29 to 3.67] during interview. Relationship with contact was a significant [AOR: 0.4, 95% CI: 0.2 to 0.9] social related factor.

**Conclusion:**

One third of tuberculosis patients adhered to household contact screening in health facilities during their treatment course. Promoting knowledge of tuberculosis in the community and continuous health education to tuberculosis patients are recommended.

## Introduction

Tuberculosis (TB) remains a major global health challenge. An estimated of 8.6 million people developed TB and 1.3 million died from the disease in 2012 [1]. As a matter of fact, the emergence of totally drug resistant Mycobacterium tuberculosis (M. tb) strains and absence of new effective vaccine made tuberculosis a threatening disease of the world [2].

Household contacts of active TB cases are at high risk of getting TB disease. TB detection rate among contacts of household members and neighbors is high especially among people exposed to case with high-grade sputum smears [3]. It is estimated that, over one year, a single pulmonary TB patient in a community can infect, on the average, 10 to 15 people she/he has contact with [4].

Patients with smear positive TB are responsible for up to 90% of the transmission occurring in the community [5]. Thus, early detection of active tuberculosis and provision of preventive therapy is essential to reduce the death rate and interrupt transmission of TB [6, 7].

Many people don't know how someone could acquire tuberculosis and also they don’t know any sign and symptom of TB [8]. This reduces effectiveness of passive case finding. So, an extensive contact investigation is essential to enhance efforts of TB control.

The Ethiopian comprehensive training manual for clinical and programmatic management of TB, Leprosy and TB/Human Immunodeficiency Virus (HIV) reiterates the need to trace and examine all close contacts of TB patients. Household contacts are screened for signs & symptoms of TB with particular attention to cough of two weeks or more duration. Other symptoms that help to identify TB suspects include fever, night sweating and weight loss. Isoniazid preventive therapy (IPT) is given for all young children (<5 years) that are household contacts of sputum smear-positive TB if they are free of TB disease [9].

Household contacts screening is not well operational zed in Amhara region. Hence, the present study was developed to investigate the adherence to household contact screening and associated factors among pulmonary tuberculosis patients in Amhara region, Ethiopia.

## Methods

### Study design, setting and participants

A cross-sectional study was conducted from April 10—June 30, 2013 in five urban districts of Amhara region. According to Amhara regional health bureau 2012 report, a total of 18,889,435 people dwell in the region; of which 87% are rural inhabitants. TB diagnostic and treatment services are free of charge in all government facilities.

All TB patients over age 18 years and presenting to the health facility and who had taken anti-TB drugs for at least one month were recruited. All of the patients were registered under the national TB control program.

### Sample size and sampling procedure

A sample size of 422 patients was obtained using the single-population proportion formula for finite populations; considering 95% confidence level, a 5% margin of error, and 10% possible non response rate. We assumed 50% of the patients to be adherent on review.

Five urban districts (Gondar, Dessie, Kombelcha, Bahirdar and Deberemarkos) were selected purposively based on geographical representation and cost implication. All eligible patients presenting during the study period were recruited.

### Data collection procedure and adherence ascertainment

Patients were interviewed using structured and pre-tested questionnaire. The research questionnaire was developed from existing literatures. Six trained nurses were participated in the data collection process. HIV status, type of pulmonary TB and adherence to household contact screening was collected from TB log book. The TB status of contacts was also taken from the TB log book.

Adherence to household contact screening before survey was assessed. Patients were asked to report the number of household contacts they brought to TB clinic for screening purpose. Their response was verified on the TB log book; as it is documentable activity. Patient was classified as adherent if he/she brought at least one household contact and otherwise non- adherent.

#### Definition of terms

Household contact: family member or any other person living and sleeping in the same house with the tuberculosis patient for at least three months before the commencement of the treatment of tuberculosis case.

Patient with sufficient knowledge on TB: a patient who answered greater than or equal to 80% of the given questions (21 items). These questions were derived from information routinely provided to patients as part of the national TB program.

### Data Processing and Analysis

Data were entered to Epi- Info version 3.5.1(Center for Disease Control and Prevention, Atlanta, GA, USA) and then analyzed using SPSS version 16(Statistical Package for Social Sciences, Chicago, IL, USA). Adherence was calculated. The mean was calculated for normally distributed data, while for skewed data the median was calculated. Both Crude Odds Ratio (COR) and Adjusted Odds Ratio (AOR) with 95% confidence interval (CI) were used to show an association between selected variables. Variables whose p-values are ≤ 0.2 during the bivariate analysis were fitted to the final multiple logistic regression model (back ward step wise) to adjust for potential confounders. In the final model, a p-value < 0.05 was considered as statistically significant.

### Ethical considerations

This study was reviewed and approved by the Ethical Review Committee of Amhara Regional Health Bureau. The Ethical Review Committee approved the oral informed consent since it was anticipated that many of the study subjects could not read and write. In addition to this, the Ethical Review Committee approved all ethical procedures, based on the awareness that the study was harmless to study subjects and the data we collected were coded and accessed only by research staff.

A support letter from local authorities and each health facility administration office was obtained. During data collection, informed oral consent was obtained from participants after they were introduced to the objective of the study and informed about their right to withdraw the interview at any time. Consenting procedures were witnessed by the tuberculosis focal person of each health facility. Personal identifiers were avoided in the questionnaire and reporting the results of the study to ensure confidentiality.

## Result

### Socio-demographic characteristics

A total of 418 pulmonary TB patients were interviewed. Of these, 164 (39.2%) were sputum smear positive and more than half of the respondents, 220 (54.5%), were males. About 337 (80.6%) respondents were from public health facilities. The median age of the respondents was 28 years; with inter quartile range of 13 years.

Majority, 331 (79.2%), of the respondents were Christians. The mean household contact size of the patients was 4 (inter quartile range = 3) and the median monthly income of respondents was 800 ET birr (43.01USD) with inter quartile range of 1100 ET birr (59.14 USD); (Table 1).

**Table 1:** Socio demographic characteristics of respondents, Amhara region, July 2013, (N = 418).

Variable	Categories	Frequency	Percent (%)	
Age	<20	32		7.7
	20–29	199		47.6
	30–39	94		22.5
	40–49	41		9.8
	50+	52		12.4
Sex	Male	228		54.5
	Female	190		45.5
Marital status	Single	195		46.7
	Married	171		40.9
	Divorced/Widowed	52		12.4
**Religion**	Christians	331		79.2
	Muslims	87		20.8
Occupation	Daily labor	82		19.6
	Merchant	73		17.5
	Student	61		14.6
	Government employee	54		12.9
	Farmer	43		10.3
	House wife	43		10.3
	No work(idle)	29		6.9
	Others*	21		5.0
	Driver /assistant driver	12		2.9
Education	No formal education	110		26.3
	Primary education	101		24.2
	Secondary education	95		22.7
	12 and above	112		26.8

* Others—carpenter, shoe shiner, commercial sex worker, mechanic

### Social related characteristics

Among 418 respondents, 80 (19.1%) had no support, 33(7.9%) had low support and 161 (38.5%) had high support from their family.

Three hundred and ninety seven (95%) respondents had good interaction with their family. Some patients (17.0%) faced stigma from the community. Three—fourth (74.9%) of participants did not have sufficient knowledge about tuberculosis.

### Health care system related characteristics

Only one third (34.2%) of the patients were very satisfied with service delivered at TB clinic. Likewise, half (51.2%) of the participants reported that it took them more than half an hour to reach the TB clinic and most of them (80.4%) travelled to and from the clinic on foot. In all the health facilities, 155 (37.1%) study subjects did not take health education from health care worker(HCW)providing them the anti-TB drugs (Table 2).

**Table 2:** Health care system related characteristics of respondents, Amhara region, July 2013(N = 418).

Variable	Categories	Number	Percentage
Type of health facility	Public	337	80.6
	Private	81	19.4
Time taken to get TB clinic	<30 minutes	204	48.8
	≥3o minutes	214	51.2
Mode of transportation to TB clinic	On foot	336	80.4
	Public transport	82	19.6
Health education by HCW	No	155	37.1
	Yes	263	62.9
Waiting time at TB clinic	<1hours	400	95.7
	1–2 hours	18	4.5
Service satisfaction	Not at all satisfied	27	6.5
	Slightly satisfied	92	22.0
	Moderately satisfied	156	37.3
	Very satisfied	143	34.2
	Extremely satisfied	0	0.0

TB- tuberculosis, HCW- Health Care Worker

### Adherence to household contact screening

The overall adherence to household contact screening in Amhara region was 33.7% (141 out of 418).The adherence level differed in HIV infected and HIV uninfected patients; being 35.3% and 34.2% respectively. This difference was not significant (p-value = 0.354).Of the total 1492 household contacts, 278(18.6%) were screened for TB disease. The overall yield of the contact screening was 6.5%, i.e., 65 TB patients/1000 screened household contacts.

### Factors associated with household contact screening adherence

In the bivariate logistic regression analysis, contact screening adherence was significantly associated with religion, family income, relationship with contact, family support, type of tuberculosis, health education by HCW and knowledge on tuberculosis.

However, in multivariate logistic regression (adjusted analysis), religion, family income, relationship with contact, health education by HCW, type of PTB and knowledge on TB were significantly and independently associated with adherence. Thus, Muslims were two times more likely to adhere to contact screening as compared to Christians (AOR = 2.4, 95%CI: (1.4, 4.2).Patients who took health education from HCW were three times (AOR = 3.2(95% CI: 1.9, 5.5) more likely to adhere to contact screening as compared to patients who did not take health education. Patients with sufficient knowledge were two times (AOR = 2.2, 95%CI: 1.3, 3.7) more likely to adhere to contact screening during the interview (Table 3).

**Table 3:** Factors associated with household contact screening adherence among TB patients using bivariate and multivariate logistic regression, Amhara region, July 2013, (N = 418).

Variable		Adherence		Crud OR	Adjusted OR	P-value
		Yes	No	(95%CI)	(95%CI)	for AOR
Religion	Christian	102	229	1	1	0.003
	Muslim	39	48	1.8 (1.1, 2.9)	2.4(1.4, 4.2)	
**Monthly income**	4.84–21.51 USD	26	91	1	1	0.013
	21.52–43.01 USD	39	65	2.1 (1.2, 3.8)	2.4 (1.2, 4.6)	0.010
	43.02–80.65 USD	41	63	2.3 (1.3, 4.1)	2.9 (1.5, 5.6)	0.002
	> 80.65 USD	35	58	2.1 (1.2, 3.9)	2.0 (1.0, 4.0)	0.053
**Relationship with contact**	Father	31	63	1	1	0.023
	Mother	52	77	1.4 (0.8, 2.4)	1.4(0.8, 2.7)	0.261
	Others	9	53	0.4 (0.2, 0.8)	0.4 (0.2, 0.9)	0.030
	Son/daughter	49	84	1.2 (0.7, 2.1)	1.0 (0.5, 1.8)	0.890
**Family support**	No support	14	66	1	1	0.068*
	Low support	15	18	3.9 (1.6, 9.6)	3.0(1.1, 8.1)	0.028
	Medium support	54	90	2.8 (1.5, 5.5)	2.5(1.2, 5.3)	0.014
	High support	58	103	2.7 (1.4, 5.1)	2.1(1.0, 4.4)	0.053
**Type of tuberculosis**	Sputum smear positive	76	88	1	1	
	Sputum smear negative	65	189	0.4 (0.3, 0.6)	0.3 (0.2, 0.5)	<0.001
**Health education by HCW**	No	27	128	1	1	
	Yes	114	149	3.6 (2.2, 5.9)	3.2 (1.9, 5.5)	<0.001
**Knowledge on TB**	Insufficient	93	220	1	1	
	Sufficient	48	57	2.0(1.3, 3.1)	2.2(1.3, 3.7)	0.004

*—marginally significant at p-value < 0.05, 1USD = 18.6Ethiopian Birr at time of study, TB- tuberculosis, OR- Odds Ratio, AOR—Adjusted Odds Ratio HCW-Health Care Worker, Others-relatives, housemaid, guard

## Discussion

Investigation of contacts of tuberculosis patients is a priority for TB control [10]. Hence, this study investigated the level and factors affecting adherence to household contact screening. Accordingly, the household contact screening adherence in this study was 33.7%. This finding is lower than the adherence level reported in Bangkok, Thailand: 52% [3]. However, it exceeds the finding from India, 14% [11].The possible explanations could be: low socio-economic status of the index cases and lack follow up to contact screening in low income countries.

As previously reported from other studies [3, 12, 13], adherence to screening of household members is affected by individual, social- cultural, accessibility and health system factors.

In this study, family income of a patient plays crucial role in adherence to household contact screening as patients with higher family income were more likely to be adherent. Similarly, in Vietnam, low income was positively associated with longer delays in tuberculosis diagnosis among women [14] and in Philippines, family income had determined intended health-seeking behavior of TB patients [15]. In Ethiopia, although TB diagnosis and treatment is being provided freely at the community level (in health centers and health posts); nevertheless 51.2% patients in this study reported the inaccessibility of tuberculosis service centers. Thus, lower income people might not afford cost of transportation to bring household contacts.

Health education by HCW was an important determinant of adherence to household contact screening as patients who took health education were three times more likely to adhere to contact screening. In our setting, the health education focuses on signs and symptoms of TB, advantage of early screening, TB infection prevention techniques and the type of person they should bring. Okuonghae in 2010[8]reported that patients who lacked information on tuberculosis cannot say much on the symptoms and signs of TB; they cannot use chronic cough as a marker for identifying a potential TB case which may lead to failure in notify the TB case to the relevant health worker.

Household contact screening was significantly lower among Christians. The belief on use of holy water (16%) and herbal medicine (1.5%) for treatment of tuberculosis among Christians, in this study, could contribute to the lower adherence. In support of this, TB patients who used holy water from orthodox churches in Tigray, Ethiopia [16] were delayed to seek modern health care. Additionally the Christians TB patients might belong to a lower socio-economic status family. As the proportion of Muslim participants in this study were small (20%), the assumption needs further investigation.

In the present study, the adherence level among HIV infected and HIV uninfected patients had no significance difference. This was consistent with previous study [16] where HIV status had no association with delayed consultation among pulmonary tuberculosis patients. However many of the HIV infected patients were expected to adhere as they have regular visits to health facility for anti retro viral therapy follows up.

Knowledge of tuberculosis patients had significant association with adherence to household contact screening. Understanding the benefit of bringing household contact to TB clinic might contribute to their adherence. Other study, in Zambia, had reported that low knowledge was preventive to treatment adherence [17]. The study sample could be possible reason of disparity.

This study has several limitations. Firstly, the study was cross-sectional and therefore no causal inferences can be made. Second, there might be social desirability bias from participants towards which they assumed good response. Thirdly, the setting was only limited to urban districts.

## Conclusion

Household contact screening adherence among tuberculosis patients was low in Amhara region. Religion, family income and relationship with contacts, type of TB, health education by HCW and knowledge on tuberculosis were significantly associated with adherence. Hence, promotion of universal knowledge of TB in the community and continuous health education to tuberculosis patients are recommended. The association between religion and adherence needs to be further investigated.
